# Validation of a sports nutrition knowledge questionnaire for athletes in the United Kingdom and Ireland

**DOI:** 10.1017/jns.2022.109

**Published:** 2023-01-06

**Authors:** Caitlin Edmonds, Ryan Tam, Sharon Madigan, Louise Gubb, Kathryn L. Beck, Janelle A. Gifford, Victoria M. Flood, Tania Prvan, Luke N. Gemming, Helen O'Connor

**Affiliations:** 1 Faculty of Medicine and Health, School of Nursing and Midwifery, The University of Sydney, Sydney, Australia; 2 Faculty of Medicine and Health, School of Health Sciences, The University of Sydney, Sydney, Australia; 3 School of Behavioural and Health Sciences, Australian Catholic University, Sydney, Australia; 4 Sport Ireland Institute, Dublin, Ireland; 5 School of Sport, Health and Applied Sciences, St Mary's University, Twickenham, London, UK; 6 School of Sport Exercise and Nutrition, Massey University, Auckland, New Zealand; 7 South Western Sydney Local Health District, Sydney, Australia; 8 Western Sydney Local Health District, Sydney, Australia; 9 School of Mathematical and Physical Sciences, Macquarie University, Sydney, Australia

**Keywords:** Construct validity, General nutrition knowledge, Internal consistency, Sports nutrition knowledge

## Abstract

Sound general and sports nutrition knowledge in athletes is essential for making appropriate dietary choices. Assessment of nutrition knowledge enables evaluation and tailoring of nutrition education. However, few well-validated tools are available to assess nutrition knowledge in athletes. The objective of the present study was to establish the validity of the Platform to Evaluate Athlete Knowledge Sports – Nutrition Questionnaire (PEAKS-NQ) for use in the United Kingdom and Irish (UK-I) athletes. To confirm content validity, twenty-three sports nutritionists (SNs) from elite, UK-I sports institutes provided feedback on the PEAKS-NQ via a modified Delphi method. After minor changes, the UK-I version of the PEAKS-NQ was administered to UK-I SN from the British Dietetic Association Sport and Exercise Nutrition Register, and elite athletes (EA) training at elite sports institutes in the UK and Ireland. Independent samples *t*-test and independent samples median tests were used to compare PEAKS-NQ total and subsection scores between EA and SN (to assess construct validity). Cronbach's alpha (good ≥ 0⋅7) was used to establish internal consistency. The SN achieved greater overall [SN (*n* 23) 92⋅3 (9⋅3) *v.* EA (*n* 154): 71⋅4 (10⋅0)%; *P* < 0⋅001] and individual section scores (*P* < 0⋅001) except Section B, Identification of Food Groups (*P* = 0⋅07). Largest knowledge differences between SN and EA were in Section D, Applied Sports Nutrition [SN: 88⋅5 (8⋅9) *v.* EA: 56⋅7 (14⋅5)%; *P* < 0⋅00]. Overall ES was large (2⋅1), with subsections ranging from 0⋅6 to 2⋅3. Cronbach's alpha was good (0⋅83). The PEAKS-NQ had good content and construct validity, supporting its use to assess nutrition knowledge of UK-I athletes.

## Introduction

Strong evidence supports the importance of nutrition for optimal athletic performance^(1)^. Despite this, athletes often have inadequate dietary intake for optimal performance and long-term health^(1–3)^. Elite sports institutes and professional sports programmes usually provide both general and sports nutrition education to support their athletes^(4,5)^. However, athletes may not apply this nutrition knowledge, as other factors such as taste, cost and convenience all influence food choice^(4,6)^. In addition, nutrition misinformation is widely disseminated in the mass media and by other athletes and coaches^(7)^. Despite these factors, sound general and sports nutrition knowledge (SNK) in athletes still underpins making appropriate dietary choices. Encouragingly, research has revealed positive correlations between greater nutrition knowledge and better dietary intake and behaviour^(8–10)^, although the literature focusing on athletes is limited^(11)^.

Nutrition education is a key strategy in supporting the health and diet of athletes; however, the level of nutrition knowledge in athletes is rarely assessed and the effectiveness of nutrition education interventions in athletes are often not evaluated^(12,13)^. Researchers have identified a lack of well-validated sports nutrition (SNK) assessment tools as a major limitation^(8,12,14,15)^. It is also recognised that nutrition knowledge, including SNK, can be a difficult construct to measure^(8,12,14,15)^. When measuring the nutrition knowledge of athletes, several factors must be considered. Elite athletes often train or compete overseas or a substantial distance away from sports institutes, making in-person or pen and paper assessment challenging. The ratio of sports nutritionists (SNs) to athletes may limit the time available to conduct individual assessments, especially in large teams of athletes^(16)^. A useful tool should exhibit the capacity to assess both general nutrition knowledge (GNK), as the foundation for optimal health, alongside sports-specific nutrition knowledge^(13,15)^.

The use of an electronic SNK tool offers several advantages. This includes the potential for immediate scoring and feedback, use of food photos or other images to assist in engagement and item comprehension, and the ability to complete the assessment virtually^(17–21)^. The potential for independent use of subsections also presents a benefit for providing assessment and feedback on specific aspects of GNK (e.g. food groups or specific nutrients) or SNK (e.g. training, competition nutrition, supplements)^(13)^. Recently, an electronic tool the Platform to Evaluate Athlete Knowledge Sports – Nutrition Questionnaire (PEAKS-NQ) was developed and validated for use in athletes in Australia and New Zealand (NZ)^(12,22)^. PEAKS-NQ is an engaging and interactive tool allowing for rapid, low-cost data capture with the capacity for immediate scoring and feedback.

Due to possible cultural differences between populations, SNK tools should be adapted and validated within the population they are deployed^(11,13,14,23)^. This enables such tools to capture differences in food group classifications, food supplies or labelling specifications. Considering the wide-spread investment of nutrition education within sporting organisations, validation of an SNK tool is likely to be a useful resource for athletes and SNs^(8,12,14,15)^. Given that the use of well-validated electronic SNK tools is limited are few well-validated SNK tools, the present study aimed to further adapt and validate the existing Platform to Evaluate Athlete Knowledge of Sports – Nutrition Questionnaire (PEAKS-NQ) for use in the United Kingdom (UK) and Ireland (I).

## Methodology

### Study design and ethics

The present study was conducted in two phases. Phase one involved the modification of an existing SNK tool developed in Australia/NZ^(12)^, the PEAKS-NQ, for use by SN and EA based in the United Kingdom/Ireland (UK-I). This phase also established face and content validity^(24)^. Phase two involved the assessment of construct validity and internal consistency in UK-I EA (August to September 2018; January to September 2019) and SN (August to September 2018).

This study was conducted according to the guidelines laid down in the Declaration of Helsinki and all procedures involving human participants were approved by the Human Ethics Committees of St Mary's University, London, UK (reference number SMEC_2018-19_016) and The University of Sydney, Sydney, Australia (protocol number 2018/311). Written informed consent was obtained from all participants.

### Development of the original PEAKS-NQ (Australia/NZ)

Focus group themes informed by SN from elite sporting institutes within Australia and NZ, sports nutrition position stands^(1,25)^ and the scientific literature were used to develop PEAKS-NQ items^(12)^. Following this, SN focus group participants took part in a modified Delphi process to provide feedback on and improve the draft items^(12)^. The final PEAKS-NQ consists of 90 items (11 demographic; 79 scored) across six sections (A–F) and a maximum score of 113: Section A – Demographics characteristics (not scored); Section B – Food Groups (13 items, 13 points); Section C – Nutrients (36 items, 41 points); Section D – Applied Sports Nutrition (14 items, 23 points); Section E – Competition Nutrition (9 items, 19 points) and Section F – Supplements and Special Concerns (7 items, 17 points). Fifty-six items (71 % total score) assessed declarative knowledge (e.g. identification of food groups) and 19 (24 % total score) assessed procedural knowledge (e.g. selection of appropriate recovery meals)^(15)^. Four items (5 %) assessed both declarative and procedural knowledge. The PEAKS-NQ was designed to be administered electronically using Filemaker Pro 12 (Claris)^(12)^. Further details of the PEAKS-NQ development, scoring and validation can be found in the publications by Tam *et al.*^(12,22)^.

### Phase 1: Adaptation of the PEAKS-NQ to UK-I setting

To ensure content validity, a cohort of experienced SN (*n* 23) from elite sports institutes in the UK-I provided feedback via a modified Delphi method on each item^(26)^. This was conducted to ensure that the items were relevant to the UK-I athlete population. Potential differences in nutrition composition (particularly fortification variations) were checked to ensure that the correct responses from the Australian/New Zealand version remained correct in the UK-I. Changes were predominantly to the demographic items (mainly different ethnicity, terminology for levels of education)^(24)^.

### Phase 2: Assessment of construct validity

Phase two, involved the assessment of construct validity through completion of the PEAKS-NQ by UK-I SN (registered with the Sports & Exercise Nutrition Register (SENr)) and athletes training at elite sports institutes in the UK-I. Construct validity ensures items are well composed to discriminate between those with different levels of SNK^(13,27,28)^. SNs were expected to perform better than athletes, given their relevant knowledge and qualifications in sports nutrition.

Permission was obtained from the British Dietetic Association (BDA) to invite SENr registrants to complete the PEAKS-NQ (UK-I version). The BDA distributed the study invitation to 250 SNs via email between July and August 2018, which included an online link for completion. Two reminder e-mails were sent to optimise participation. A convenience sample of elite athletes at three sports institutes in the UK-I were invited by institute staff to complete the PEAKS-NQ (UK-I version) via an online link. These scores provided a comparative benchmark with the SN.

### Statistical analysis

Statistical analysis was conducted using IBM SPSS Statistics for Windows (Version 24.0) and Minitab 18. Statistical significance was set at *P* < 0⋅05. The Ryan-Joiner Normality Test was used to assess normality of the data. Data are presented as total scores and percentages [mean (standard deviation)] and frequency data for categorical variables. The *χ*^2^ test, Fisher's exact test (categorical data), independent samples *t*-test and independent sample median test (continuous data) were used to investigate differences in PEAKS-NQ demographic characteristics (Section A) and PEAKS-NQ Section B–F and total scores between EA and SN. A general linear model incorporating potential confounders (age, education, sport category and sport level) was fitted to compare total scores for male and female EA. Internal consistency (the inter-relatedness of items within a questionnaire; or a measure of how well items in a questionnaire measure the same concept or construct) was calculated for the PEAKS-NQ total score and each subsection using Cronbach's alpha (good >0⋅7)^(10,29)^.

The anticipated effect size for the total score, calculated using the Hedges’ *g* method, was 2⋅1. The sample size was calculated to detect a 10 % difference in the total score at 80 % power and two-sided significance level 0⋅05. To achieve this, seventeen participants were required in each group, assuming a standard deviation of 10 % in the two-sample *t*-test. The standard deviation of 10 % was consistent with a pilot study by Gubb, Solanas^(24)^.

## Results

### Demographic characteristics

Thirty-five SNs commenced the PEAKS-NQ with 23 (66 %) completing all sections of PEAKS-NQ. A total of 316 EA commenced the PEAKS-NQ with 154 (49 %) completing PEAKS-NQ. The SNs were significantly older than the EA (*P* < 0⋅001). The SNs were all university educated, whereas only 34⋅4 % of the EA were university educated (*P* < 0⋅001). There was a higher proportion of endurance sports than other sports represented in the EA cohort (*P* = 0⋅01), and the majority were competing at open international level (Table 1).

**Table 1.** Participant demographic characteristics – PEAKS-NQ (UK-I) (*n* 177)

Demographic characteristics	Elite Athlete (*n*)	Elite Athlete (%)	Sports Nutritionist (*n*)	Sports Nutritionist (%)	*P*-value
*n* (%)	154	87	23	13	
Age (years)^a^	21⋅6	(5⋅4)	35⋅8	(10⋅3)	<0⋅001
Sex^b^					0⋅44
Male	67	44	12	52	
Female	87	56	11	48	
Ethnicity^c^					0⋅048
British/Irish	150	97	20	87	
Other	4	3	3	13	
Education^c^					<0⋅001
<Year 12	13	8	0	0	
Year 12–13	35	23	0	0	
Vocational training	3	2	0	0	
University	53	34	23	100	
Other	50	33	0	0	
Sport category^b^^,^^d^					0⋅01
High intensity/intermittent	61	40	NA		
Endurance	93	60			
Competition level^b^					
Open international	82	53	NA		<0⋅001
Junior international	43	28			
School/regional/national	29	19			

PEAKS-NQ (UK-I), Platform to Evaluate Athlete Knowledge Sports – Nutrition Questionnaire (United Kingdom and Ireland).

a Mean (standard deviation), calculated using an independent samples *t*-test with a Box Cox transformation of age.

b *n* (%), *χ*^2^ goodness-of-fit test.

c *n* (%), Fisher's exact test.

d Sport category – High intensity/intermittent sports: combat sport (boxing and martial arts), track and field (sprinting and jumping), sailing and team sports. Endurance Sports: walking, running, swimming, rowing, cycling.

### Performance on the PEAKS-NQ (UK-I)

Performance on the PEAKS-NQ for EA and SN is summarised in Table 2. As expected, the SN scored higher overall [SN 91⋅3 % (3⋅5 %) *v*. EA 71⋅4 % (10⋅0 %); *P* < 0⋅001]. The SN scored above 88 % on all sections. The highest scoring sections were Section B (Food Groups), Section C (Nutrients) and Section F (Supplements and Sports Nutrition) for both SN and EA. The lowest scoring sections for both SN and EA were Section D (Applied Sports Nutrition) and E (Competition Nutrition). SN scored significantly higher than EA on each of the subsections of the PEAKS-NQ (*P* < 0⋅001), except for section B (Food Groups), where there was no significant difference between the groups. Internal consistency of the tool was good, with a Cronbach's alpha of 0⋅83 overall. Cronbach alpha scores for sections B, C, D, E and F were 0⋅20, 0⋅67, 0⋅52, 0⋅53 and 0⋅58, respectively.

**Table 2.** PEAKS-NQ results for Elite Athletes and Sports Nutritionists expressed as raw scores and percentage

Knowledge domains	Elite Athlete (*n* 154)				Sports Nutritionist (*n* 23)				*P*
	Raw score mean	Raw score (sd)	% score mean	% score (sd)	Raw score mean	Raw score (sd)	% score mean	% score (sd)	
Section B: 13 points									
Food groups^a^	11⋅9	(1⋅0)	91⋅5	(7⋅8)	12⋅4	(0⋅8)	95⋅7	(5⋅6)	0⋅07
Section C: 41 points									
Nutrients^a^	31⋅3	(3⋅9)	76⋅3	(9⋅5)	37⋅9	(1⋅7)	92⋅5	(4⋅1)	<0⋅001
Section D: 23 points									
Applied sports nutrition^a^	13⋅03	(3⋅3)	56⋅7	(14⋅5)	20⋅3	(2⋅0)	88⋅5	(8⋅9)	<0⋅001
Section E: 19 points									
Competition nutrition^a^	11⋅9	(3⋅0)	62⋅3	(15⋅8)	16⋅8	(1⋅0)	88⋅6	(5⋅4)	<0⋅001
Section F: 17 points									
Supplements and sports nutrition^a^	12⋅5	(3⋅5)	73⋅5	(20⋅5)	15⋅7	(1⋅6)	92⋅3	(9⋅3)	<0⋅001
Total score: 113 points^b^	80⋅7	(11⋅3)	71⋅4	(10⋅0)	103⋅2	(4⋅0)	91⋅3	(3⋅5)	<0⋅001

PEAKS-NQ, Platform to Evaluate Athlete Knowledge Sports – Nutrition Questionnaire.

a Significant difference calculated using an independent samples median *t*-test.

b Significant difference calculated using an independent samples *t*-test with a Box Cox transformation.

Among EA, there were no significant differences in scores between males and females, different competition levels or sport categories, except for Section D (Applied Sports Nutrition), where high intensity/intermittent EA scored better than endurance EA. There were no significant differences in scores between education levels, except for Section E (Competition Nutrition) where university educated EA scored significantly higher [68⋅0 % (13⋅6 %)] than those with lower levels of education [school: 60⋅3 % (16⋅5 %); other 59⋅5 % (16⋅2 %)]. After incorporating potentially confounding factors into a general linear model; adjusting for age, education, sport category and sport level, female EA scored higher than males for total score by 3⋅5 % on average (*P* = 0⋅03; Table 3).

**Table 3.** PEAKS-NQ total and subsection scores for confounding variables in Elite Athletes

Confounding variable	Subsection and total scores as a % [mean (sd)]											
	B		C		D		E		F		Totals	
Sex												
Female	92⋅5	(7⋅7)	78⋅5	(8⋅3)	57⋅8	(13⋅5)	63⋅6	(17⋅9)	76⋅9	(20⋅0)	73⋅1	(9⋅2)
Male	90⋅9	(7⋅8)	73⋅4	(10⋅2)	55⋅2	(15⋅6)	61⋅8	(18⋅5)	69⋅3	(20⋅6)	69⋅2	(10⋅5)
*P*-value^a^		0⋅22		0⋅07		0⋅97		0⋅87		0⋅08		0⋅13
Education												
School	91⋅5	(6⋅8)	75⋅3	(8⋅7)	55⋅4	(15⋅3)	60⋅3	(16⋅5)	69⋅0	(22⋅4)	69⋅6	(10⋅4)
University	92⋅4	(7⋅1)	77⋅2	(9⋅0)	59⋅4	(14⋅6)	68⋅0	(13⋅6)	76⋅4	(19⋅4)	73⋅7	(9⋅2)
Other	91⋅4	(9⋅3)	76⋅1	(10⋅7)	54⋅7	(13⋅3)	59⋅5	(16⋅2)	74⋅8	(19⋅4)	70⋅5	(10⋅1)
*P*-value^a^		0⋅39		0⋅12		0⋅37		**0⋅008**		0⋅47		0⋅13
Sport category												
High intensity/intermittent	91⋅7	(8⋅0)	75⋅8	(10⋅0)	58⋅7	(14⋅8)	63⋅3	(16⋅1)	69⋅3	(22⋅1)	71⋅1	(10⋅6)
Endurance	91⋅9	(7⋅6)	76⋅6	(9⋅2)	55⋅4	(14⋅2)	62⋅5	(15⋅7)	76⋅3	(19⋅0)	71⋅6	(9⋅6)
*P*-value^a^		0⋅86		0⋅99		**0⋅011**		0⋅65		0⋅053		0⋅64
Competition level												
Open international	11⋅8	(1⋅0)	30⋅9	(3⋅7)	13⋅5	(3⋅4)	11⋅9	(2⋅9)	12⋅4	(3⋅8)	80⋅5	(11⋅6)
Junior international	12⋅0	(1⋅1)	31⋅2	(4⋅3)	12⋅4	(3⋅2)	12⋅2	(3⋅5)	12⋅8	(2⋅4)	80⋅6	(11⋅9)
School/regional/national	12⋅1	(1⋅0)	32⋅4	(3⋅7)	12⋅7	(3⋅2)	11⋅6	(2⋅6)	12⋅5	(2⋅4)	81⋅3	(9⋅6)
*P*-value^a^		0⋅10		0⋅24		0⋅10		0⋅35		0⋅61		0⋅93

a Independent samples median test.

The bolded values provide the significance levels.

## Discussion

Following adaptation of the PEAKS-NQ for the UK and Ireland, the results demonstrate substantial differences in GNK (aside from knowledge of food groups) and SNK between EA and SN, supporting the construct validity of the PEAKS-NQ. Internal consistency of the overall PEAKS-NQ was also high (0⋅83). After controlling for potential confounders, female athletes scored slightly but statistically significantly higher than male athletes.

Criticisms of existing tools highlight the lack of validation and thus ability to confidently use in practice^(8,12,13,15)^. The PEAKS-NQ was evaluated through a modified Delphi process with experienced UK-I SN supporting its relevance to a UK-I population^(24)^. The scores achieved by the SN and EA cohorts confirm the construct validity of the tool, in that it successfully differentiates between groups with varying levels of nutrition knowledge^(10)^. As expected, SN performed consistently higher than EA in both total score and subsections, except Section B (Identifying Food Groups). Categorising foods into food groups is declarative knowledge (factual information stored in the memory) and included in the national curriculum of local authority-maintained schools in the UK-I^(30)^, which may help to explain why differences were not observed between SN and EA. Both the SN and EA achieved their highest scores in Section B (Food Groups), Section C (Nutrients) and Section F (Supplements and Sports Nutrition). Relatively high scores achieved in Supplements and Sports Nutrition in the EA cohort, similar to Torres-McGehee, Pritchett^(31)^, may reflect the high calibre of athletes recruited, and also the focus on educating athletes about safe supplement use^(12,31)^.

EA had low scores for Section D (Applied Sports Nutrition) and E (Competition Nutrition). These domains had the largest proportion of procedural knowledge-based questions, requiring practical application of nutrition knowledge. Section D saw the largest knowledge difference between EA and SN, which is not unreasonable given SN are highly trained in applying specific SNK. It is also frequently reported that athletes consume a diet inadequate for achieving and maintaining both optimal health and performance^(1–3)^. This suggests a disconnect in the translation of nutrition knowledge to appropriate dietary selection in athletes. Previous evaluation of athlete nutrition knowledge, suggests athletes possess basic nutrition, yet procedural nutrition knowledge is weaker^(9,32)^. These data are consistent with previous studies validating knowledge assessment tools, which report ‘nutrition experts’ consistently perform stronger than other cohorts^(9,13,14)^. The PEAKS-NQ demonstrated overall good internal consistency, with an overall Cronbach's alpha of 0⋅83. However, individual sections had lower Cronbach alpha scores. In Section B, many athletes scored correctly on all or some items, meaning that the variance was zero or low, contributing to a lower internal consistency. The lower number of questions within individual sections (B, D, E and F) may have also contributed to lower Cronbach alpha scores^(29)^.

The sample of SN were recruited from the SENr, which includes highly qualified sports nutritionists from the UK-I. Although only a small sample, participants achieved high subsection and overall scores, indicative of their expertise. This confirms they were an appropriate cohort to inform construct validity when compared with EA. SN scores were almost identical to another study utilising PEAKS-NQ, where it was reported that Australian sports dietitians had an overall score of 91⋅1 % (6⋅4 %)^(22)^. The large EA cohort represented a variety of sports, with the majority competing at open international level and with various stages of education. The SN cohort were significantly older than the EA, which is expected given all SN had completed a university degree along with career experience. A key component of thorough validation is the specificity to the population intended for use^(11,13,14,23)^. The spread of demographics in this validation study speaks to the usefulness and reliability of the PEAKS-NQ for use in the UK-I.

There were no differences in total scores when individually comparing sex, education, competition level or sport type within the EA cohort, suggesting differences in scores were unlikely to be attributable to participant characteristics^(8,9)^. When adjusting for the confounding variables (age, education, sport type and sport calibre), sex was found to influence the total score, with females performing slightly better than males (3⋅5 %). Previous studies have reported similar results, with females performing better than males^(8,10,32)^. For example, using the same tool, Tam *et al.*^(32)^ found female athletes scored 5⋅7 % higher than male athletes, while Wardle *et al.*^(10)^ observed females from the general population scored 4⋅4 % higher than males. There have been various reasons suggested for this trend, including less interest among males in nutrition and also important life stages for women producing greater concern for nutrition (moving away from home, marriage, pregnancy, etc.)^(33,34)^. However, findings suggesting an influence for sex are contrasted by Trakman, Forsyth^(14)^'s review, which reported ten of fifteen studies found no significant differences in nutrition knowledge between males and females. Education had no significant influence on scores, except for Section E (Competition Nutrition), where university educated EA performed better than those of lower educational attainment. This is in contrast with previous findings which have suggested those with higher education consistently perform better^(10,13,23)^. The lack of difference in overall score between education groups may be again a reflection of the high calibre of athletes recruited for this study, who are more likely to be exposed to nutrition education through their sport compared with developmental athletes. The EA in this study scored 71⋅4 % (10⋅0 %) which is higher than New Zealand developmental athletes who scored 67⋅1 % (10⋅5 %) on PEAKS-NQ^(22)^. Performance within the EA cohort when comparing sport categories indicated no significant difference in overall score, except for Section D (Applied Sports Nutrition) which identified a slight but significantly higher score in the high intensity/intermittent sports.

Despite the validity and internal consistency of the PEAKS-NQ, there are several limitations to this research. Convenience sampling was utilised for participant recruitment, which may have impacted the size and representativeness of the sample. This, in turn, may affect the generalisability of the tool for use with a wider athlete population. A more suitable recruitment method would be to collect a stratified, random sample of athletes, ensuring recruitment from a wide range of demographics and sport calibre and categories. Additionally, due to the small SN cohort, findings may not be truly representative of SN in the UK-I. Despite this, SN scored well, similar to a large cohort of Australian Sports Dietitians utilising the same tool^(22)^. The SN and EA were predominantly of British/Irish ethnicity, which may not necessarily be representative of the diverse population in the UK-I. Therefore, when used within a more diverse range of ethnicities, the utility of the PEAKS-NQ needs to be further determined.

There were many strengths to the present study which contribute to the validation and utility of this tool for assessing nutrition knowledge in UK-I EA. Prior to validation, the tool underwent thorough development and evaluation to inform face and content validity^(12)^. In development, highly qualified sports dietitians participated in focus groups and ongoing consultation to further refine the tool. The items have also been previously evaluated in developmental athletes^(22)^. For adaptation of the tool for use in the UK-I, sports nutritionists were consulted through a modified Delphi process to provide thorough feedback and ensure appropriateness of the tool for this demographic. The criterion (SN) group used as the benchmark to inform construct validity was selected as highly trained professionals. The athlete cohort were sourced from elite sports institutes. These groups provided comparative data for the purpose of this study. Importantly for validation, a balanced sample of males and females, from a wide range of sports, were represented in the EA sample, which supports the generalisability and usability of the tool. The online administration was anecdotally reported to be easy, practical and free of any technical issues, which is critical for utility of the tool.

The practicality of a knowledge assessment tool is essential when working with elite athletes due to often having limited time availability and the highly dynamic nature of the work. Given the unique demands of elite sport and large number of athletes within a sport or institute requiring SN input, a flexible and practical tool is highly advantageous. The PEAKS-NQ takes approximately 15 min to complete and offers a thorough assessment of both GNK and SNK, in which the electronic platform allows for immediate scoring and feedback. Beyond informing on athlete nutrition knowledge, this tool has the potential to be a useful tool for evaluating the effectiveness of nutrition interventions. Due to the high calibre of athletes recruited in this study, it is difficult to suggest benchmark levels of knowledge or a ‘cut off’ for adequate knowledge for athletes of varying calibre. Previous studies have nominally suggested scores greater than 75 % indicate adequate or even excellent knowledge^(13,32)^. A score of 90 % as achieved by the sports dietitians in this study is suggested as a logical cut-off for ‘excellent’ knowledge, while the elite athletes scored on average 71 % indicating somewhat ‘average’ knowledge. With further validation papers utilising the PEAKS-NQ and higher completion numbers, knowledge benchmarks will likely be assigned to provide guidance on adequacy of performance.

## Conclusion

In conclusion, the present study confirmed the validity of the PEAKS-NQ tool for use in assessment of SNK in UK-I athletes. The findings suggest that the PEAKS-NQ successfully differentiates level of GNK and SNK (aside from knowledge of food groups) between Elite Athletes and Sports Nutritionists. These results confirm the construct validity of PEAKS-NQ, supporting confident use for evaluating the knowledge of athletes within a UK-I population. The assessment of nutrition knowledge in athletes is highly advantageous for creating more specific and effective nutrition interventions, which will in turn support dietary intake and optimal performance of athletes. The present study confirms that the PEAKS-NQ is a valid and practical tool for assessing nutrition knowledge in UK-I athletes.
